# Adverse Childhood Experiences Distinguish Violent Juvenile Sexual Offenders’ Victim Typologies

**DOI:** 10.3390/ijerph182111345

**Published:** 2021-10-28

**Authors:** Michael T. Baglivio, Kevin T. Wolff

**Affiliations:** 1 Youth Opportunity Investments, LLC., Carmel, IN 46032, USA; 2 Department of Criminology, University of South Florida, Tampa, FL 33620, USA; 3 Department of Criminal Justice, John Jay College of Criminal Justice, City University of New York, New York, NY 10019, USA; KWolff@jjay.cuny.edu

**Keywords:** adverse childhood experiences (ACE), victim typologies, violent juvenile sexual offending, latent class analysis, victim–offender relationship

## Abstract

Juvenile perpetrators account for over 25% of all sexual offenses, and over one-third of such offenses are against victims under the age of 18. Given empirical connections between adverse childhood experience (ACE) exposure and perpetration of violence, we create victim typologies based on the juveniles’ relationship to their victims among 5539 justice-involved adolescents who have committed violent against-person sexual felonies. Multinomial logistic regression is used to assess which covariates, including individual ACE exposures and cumulative traumatic exposures, are associated with victim typologies. This approach allows for better targeting of violence prevention efforts, as a more nuanced understanding of the increased likelihood to victimize specific victim groups lends to potential differences in treatment provision, beyond simplistic findings regarding ACE exposure increasing offending. Results indicate five classes of victim types, ranging from a low of 6.4%, with primarily strangers as victims, to 31.3%, with predominately acquaintances as victims, and only 12.9% with a diverse array of relationships to victims. Importantly, many demographic and individual risk factors, and specific traumatic exposures were related to victimizing one’s sibling, while cumulative trauma as measured by an ACE score decreased the likelihood of victimizing classmates, while increasing the likelihood of victimizing siblings and other relatives compared to victimizing acquaintances.

## 1. Introduction

Juveniles who commit sexual offenses commit approximately one-in-four of all sexual offenses, and over one-third of all sexual offenses against juveniles [1]. Approximately 57% of the victims of sexual assaults committed by juveniles were younger than the age of 12, with 16% of those victims between ages 3 to 5, while less than 4% of the victims were 18 years of age or older [2]. The heterogeneity among juveniles who sexually offend is now well elucidated [3,4,5,6,7,8,9,10,11]. Typologies of juveniles who sexually offend are most often distinguished, at least in part, by the age of their victims: differentiating between those offending against peers or adults versus those against children [12,13]. Most often, however, the victim types in prior work have been based on the victim(s) of a single presenting offense, rather than consideration of every known victim over time. This approach may over-presume heterogeneity, whereas the entirety of one’s sexual offending may demonstrate a lack of specialization in victim types (even, for example, if young children are always the victim, whether those children are always family members, strangers, etc., is a relevant distinction). One rather consistent finding among studies examining predictors of who engages in juvenile sexual offending, including among adolescents with justice system involvement, is the offending youth’s own victimization experiences of various types of childhood abuse and household dysfunctions [1,10,14]. While sexual abuse most strongly contrasts sexual offending versus nonsexual offending among youth with juvenile justice system involvement [14], other prominent traumatic exposures predictive of juvenile sexual offending have included physical abuse, parental abandonment, emotional abuse, witnessing household violence, and substance abuse problems among members of the household [4,10,14]. Among adolescents involved in the juvenile justice system in Florida (the current study’s focus), those with sexual offending arrests had 275% greater odds of having a history of sexual abuse than those without sexual offenses [14]. Further, DeLisi and colleagues [15] demonstrated that cumulative ACEs strongly and positively associated with sexual offending among incarcerated juveniles. Hunter and colleagues [16] demonstrated childhood sexual victimization by a male nonrelative was associated with sexual offending against a male child, demonstrating certain abuse types may be more indicative of who a victimized youth may later sexually offend against. 

While historically the independent effects of individual indicators of abuse, neglect, and household dysfunctions were examined among studies either distinguishing juveniles who sexually offend or developing typologies among those adolescents that have sexually offended [4,5,10,14], recent work has clearly elucidated the interrelatedness of adverse childhood experiences, that such exposures tend to co-occur, and that experiencing one type of exposure dramatically increases the odds of experiencing other types [17,18]. As such, it is argued essential to also examine cumulative traumatic exposure rather than only the independent effects of each exposure type [19] vis à vis an adverse childhood experience (ACE) score [20]. While not yet explored among typologies of the victims that juveniles sexually offend against, a growing body of work has indicated that specific traumatic exposure types should be examined as differentiators [21,22,23]. Notably, a prior study leveraged latent class analysis to explore whether cumulative and specific ACE exposures differentiate juveniles who commit nonsexual violent felonies based on the relationship between these youth and the victims of their violent offenses [21]. Findings demonstrated cumulative ACEs decrease the odds of violent (nonsexual) offending against strangers but increase the likelihood of victimizing family, those in positions of authority, and having multiple victim types. Further, household mental health problems and incarceration history of household members had the most substantial impact among all ACE exposure types, with household incarceration the only ACE to increase offending against strangers [21]. That study demonstrated that both sexual abuse history and witnessing family violence increased the odds the juvenile would (nonsexually) violently victimize family members, while decreasing the likelihood of nonsexual violence against strangers. The current study aims to replicate that analysis among juveniles who violently sexually offend, while advancing methodologically to consider cumulative ACE exposure as well as the independent effects of each individual ACE, to ascertain whether ACEs distinguish among such adolescents based upon typologies of the victims of these youth’s sexual offending. 

Unfortunately, prior studies of juveniles who sexually offend have not yet considered the entirety of all known victims and have used constellations of victim types to develop typologies. Addressing this rather substantive gap, the current study considers all known victims of all of a given youth’s sexual offenses through to the age of eighteen. Given the prominence of traumatic exposure histories among juveniles who sexually offend, the current study aims to examine whether specific adverse childhood experiences and/or the cumulative ACE score can be leveraged to predict the victim types of youth who violently sexually offend based on the relationship between the offending youth and their victim(s). Unfortunately, the majority of work exploring typologies of juveniles who sexually offend has focused on very small samples (46 of the 59 studies in the Seto and Lalumiére meta-analysis examined fewer than 60, predominately male, juveniles [10]). Further, a large study of Florida juvenile offenders included both nonviolent and violent offenses [14]. The current study is unique in that it includes over 5500 juveniles, all of which are justice-involved, and all of which have committed violent sexual felonies. Further, the current work fills critical gaps in prior work by (1) including the entirety of all victims of all known violent sexual offenses through to the age of eighteen of each youth, and (2) examining whether indicators of ACE and/or cumulative ACE exposure distinguish victim type typologies.

### The Current Study

The current study examined whether specific ACEs and/or cumulative ACE exposure distinguish between typologies of adolescents who commit violent sexual offenses with respect to the relationships between those youth and their victims. As such, the study aimed to establish a victim–offender relationship link between the victims of violent sexual offending and the characteristics of the juveniles who target them. Importantly, advancing from prior work, the current study includes the victim types from each youth’s entire juvenile offending up to age 18, meaning that each victim of each violent sexual offense is considered. Focusing on the abuse, neglect, and household dysfunction exposures of the juveniles engaged in violent sexual offending uncovers potential precursors to targeting specific victim types of such offending and enhances crime prevention and targeted-treatment intervention strategies. Determining whether heterogeneity exists allows treatment to be better targeted, as victimizing one’s parents, friends, teachers, or strangers may necessitate individualized prevention and intervention strategies. Importantly, with respect to policy and juvenile justice strategies, these traumatic exposures are both preventable and treatable.

## 2. Materials and Methods

### 2.1. Participants and Data

All youth with a history of juvenile justice system involvement in Florida who turned 18 years of age (the age of majority in Florida) between 1 January 2007 and 31 December 2017 who committed a violent felony sexual offense by age 18 were eligible for inclusion in the study. The sample was then limited to those youth that were assessed using the Florida Department of Juvenile Justice’s (FDJJ) risk/need assessment, the Positive Achievement Change Tool (PACT; described below). Only youth assessed using the PACT full assessment were included, as only the full assessment contains all requisite items to capture all ten ACEs. This strategy resulted in a final sample of 5539 juveniles who violently sexually offended against others by their 18th birthday. 

Data were garnered from the FDJJ information system, which maintains complete demographic, offense, justice system placement, and risk/needs assessment information on all individuals arrested in Florida under the age of 18. While the PACT assessment is the primary data source, the victim measures were gleaned from a series of “flags” within the information system indicating who the victim of juveniles’ offenses were. Youth formally processed into the juvenile justice system are administered the PACT assessment, which has two versions: a prescreen and a full assessment. Both provide an identical overall risk to reoffend classification (low, moderate, mod–high, or high risk-to-reoffend); the full assessment additionally provides risk and protective domain scores (criminal history, school, free time, employment, relationships, family, substance use, mental health, attitudes, aggression, and social skills). Further, the full assessment contains items that enable a recreation of the ten predominant ACE exposures. Extensive prior work has leveraged the PACT ACE score construction (e.g., [24,25,26,27]), including in examining ACEs among those adolescents who sexually offend [28]. The PACT is conducted as a semi-structured interview, where most PACT items are self-reported and corroborated when possible (with parents/guardians, child welfare workers, or teachers/school records), with the exception of criminal history items (prior offending) which are automated from the FDJJ information system.

The data supporting the findings of the current study are available from the Florida Department of Juvenile Justice (data source), though restrictions apply to the availability of these data, which were under license for this study, and therefore are not publicly available.

### 2.2. Measures

The dependent measure regarding the relationship between the youth committing the violent sexual offense(s) and his/her victim (described below) was extracted directly from the FDJJ information system, as were demographic measures. All other covariates, including the ACE indicators and ACE score, were gathered from the youth’s first ever PACT assessment (the earliest arrest for which a full PACT assessment was completed). Most PACT items are self-reported and corroborated, when possible (with parents/guardians, child welfare workers, or teachers/school records), with the exception of criminal history items (prior offending) which are automated from the FDJJ information system. All arrests of every juvenile under the age of 18 are maintained in the FDJJ information system, which allows automation into the PACT assessment with respect to prior offending and severity of offending.

#### 2.2.1. Victim–Offender Relationship

The dependent measure assessed the relationships between the offending juvenile and his/her victims of violent (against-person) felony sexual offending. Data included each victim indicated within the FDJJ information system for each violent sexual offense each youth committed prior to age 18. Eleven victim–offender relationships classifications were made for each offense: strangers, acquaintances, schoolmates, neighbors, friends, non-sibling relatives, juvenile justice system youth, authority figures (including juvenile justice workers), siblings, romantic partner/ex-partner, and the youth’s own child. Table 1 provides the proportion of youth that commit a violent sexual offense against each victim type, ranging from a low of 2% for the youth’s own child to high of 45.6% for acquaintance victims.

#### 2.2.2. Key Independent Measure: Adverse Childhood Experiences (ACE)

While created to assess juveniles’ overall risk to commit delinquent or criminal offenses, the PACT assessment contains items which encompass the ten specific ACE items identified by the CDC (see [20]). The ACE scale includes five child maltreatments and five types of household dysfunction. The following ten ACE indicators were included and coded dichotomously (yes = 1, no = 0): (1) Emotional abuse: Parents/caretakers were hostile, berating, and/or belittling to youth; (2) Physical abuse: The youth reported being a victim of physical abuse by a family member; (3) Sexual abuse: The youth reported being the victim of sexual abuse/rape; (4) Emotional neglect: The youth reported no support network, little or no willingness to support the youth by the family, youth does not feel close to any family member; (5) Physical neglect: The youth has a history of being a victim of neglect (includes a negligent or dangerous act or omission that constitutes a clear and present danger to the child’s health, welfare, or safety, such as: failure to provide food, shelter, clothing, nurturing, or health care); (6) Family violence: The level of conflict between parents included verbal intimidation, yelling, heated arguments, threats of physical abuse, domestic violence, or the youth has witnessed violence at home or in a foster/group home; (7) Household substance abuse: History of parents and/or siblings in the household abusing alcohol or drugs; (8) Household mental illness: History of parents and/or siblings in the household includes mental health problems; (9) Parental separation/divorce: Youth does not live with both mother and father; and (10) Incarceration of household member: There is a jail or prison history of family members. 

ACE exposures were summed for a cumulative ACE score, ranging from 0 (no exposures) to 10 (exposed to each of the ten ACE indicators). For additional clarity, ACEs were assessed at the time of first arrest of the juvenile, where each ACE indicator is self-reported by the youth (consistent with the original ACE Study; [20]), as well as corroborated child welfare records (to which the PACT assessors have access). The youth’s self-reported affirmative response, as well as instances in which child welfare records indicate abuse or exposure are counted as an endorsement of each ACE item. Instances in which child welfare investigations led to decisive findings that the maltreatment did not occur are counted as a “no” for a given ACE indicator, and inconclusive child welfare investigations are captured according to the youth’s self-reported response. As shown in Table 1, prevalence rates for individual ACE indicators ranged from a low of 5.8% for household mental illness to a high of 85.1% for parental separation/divorce, with an average ACE score of 2.76 for the sample presently understudy.

#### 2.2.3. Out-of-Home Child Welfare Placements

This measure distinguishes between youth as either never having or having any out-of-home child welfare placements (yes = 1). Of note, 20.1% of the sample had at least one out-of-home child welfare system placement.

#### 2.2.4. Mental Health Problem

Youth with and without a formal mental health diagnosis were distinguished (20.6% had such a diagnosis). Of note, diagnosis must have been made by a qualified professional (e.g., psychologist, licensed mental health counselor). Mental health problems included schizophrenia, bipolar, mood, thought, personality, and adjustment disorders. Per FDJJ policy, conduct disorder, oppositional defiant, Attention Deficit Disorder/Attention Deficit Hyperactivity Disorder (ADD/ADHD), and substance disorders were excluded.

#### 2.2.5. Current Alcohol Use

Whether the youth was currently using alcohol (within the past 4 weeks) at the time of the first PACT full assessment was captured by distinguishing youth without such use from those with current use and those whose alcohol use is causing problems across life domains such as school, family, health, peer associations, or criminal behavior (coded 0–2, respectively). The juvenile probation officer (conducting the PACT assessment) made the determination that alcohol use was causing problems based on self-report from the youth as well as arrest reports, and collateral contact (parents, teachers) where possible. If the arrest report indicated substance use at the time of the offense, the youth indicated skipping school to drink alcohol, not getting to school or work on time (or going under the influence), if the youth indicated prosocial youth do not associate with him/her due to use, or if the youth (or parents) indicated his/her alcohol use was the topic of arguments or family issues, such use would be considered to cause problems.

#### 2.2.6. Current Drug Use

Similarly, whether the youth was currently using drugs (within the past 4 weeks) at the time of the first PACT full assessment was captured by distinguishing youth without such use from those with current use and those whose drug use is causing problems across life domains such as school, family, health, peer associations, or criminal behavior (coded 0–2, respectively).

#### 2.2.7. Peer Associations

Peer association distinguished youth as having prosocial and antisocial peers (mixed peer group), exclusively antisocial peers (lack of prosocial peers), or self-reported gang involvement (coded 0–2, respectively). At the time of the first PACT full assessment 3.2% self-reported gang involvement. 

#### 2.2.8. Difficult Temperament

An index of difficult temperament was created through a combination of 10 PACT items (α = 0.871). The resulting temperament construct encapsulated both effortful control and negative emotionality items [29], coded such that lower effortful control and higher negative emotionality equated to a more difficult temperament. Items included impulsivity, belief in the ability to control one’s own antisocial behavior, empathy, respect for property, respect for authority, attitude toward law-abiding behavior, frustration tolerance, hostile interpretation of other’s intent/actions, belief in using verbal aggression, and belief in using physical aggression.

Specifically, *impulsivity* distinguished youth who usually think before acting, those exhibiting some self-control, impulsive youth, and highly impulsive youth, coded 1–4, respectively, with higher values indicating greater impulsivity. *Belief in the ability to control one’s own antisocial behavior* distinguished those who believe he/she can avoid or stop antisocial behavior, those who somewhat believe so, and those who believe his/her antisocial behavior is out of his/her control (coded 1–3, respectively, with higher values indicating less control of antisocial behavior). *Empathy* separated those with empathy for his/her victims, those with some empathy, and those that do not have/express empathy for victims of his/her criminal behavior (coded 1–3, with higher values indicating less empathy for victims). *Respect for property* distinguished those who respect other’s property, those who respect personal but not public property, those with conditional respect (“if they are stupid enough to leave it out, they deserve losing it”) and those who have no respect for others property (coded 1–4, with higher values indicating less respect for property). The *respect for authority* item contains options for respecting most authority figures, does not respect, resents most authority, and those that defy or are hostile toward most authority figures (coded 1–4, with higher values indicating less respect for authority). *Attitude toward law-abiding behavior* distinguishes youth who abide by conventions/values, those that believe such values sometimes apply, those that do not believe in conventions/values, and those that resent or are hostile to responsible behavior (coded 1–4, with higher values indicated a less positive attitude toward law-abiding behavior). The *frustration tolerance* PACT item distinguishes among those that rarely get upset over small things, those that sometimes get upset, and those that often get upset over small things or have temper tantrums (coded 1–3, with higher values indicating less tolerance for frustration). *Hostile interpretation of others’ intent/actions* separates primarily positive view of the intentions of others, primarily negative view, or primarily hostile view of the intentions of others (coded 1–3, with higher values indicating a more hostile interpretation). *Verbal aggression* agreement distinguishes those that believe such aggression is rarely appropriate, sometimes appropriate, or often appropriate (coded 1–3, with higher values indicating more appropriateness attributed to using verbal aggression). Similarly, *physical aggression* agreement captures the belief that such aggression is never appropriate, rarely appropriate, sometimes appropriate, or often appropriate (coded 1–4, with higher values indicating greater belief that physical aggression is appropriate to resolve disagreements). 

#### 2.2.9. Exposure to Community Violence

Youth are distinguished by whether they have a history of witnessing violence in the community (yes = 1), with 42% of the sample having such a history (see Table 1).

#### 2.2.10. Demographics

Demographic measures included gender (male = 1, 94.8%), race (Black = 1; 45.8%), ethnicity (Hispanic = 1; 12.6%), and age at time of first PACT full assessment (continuous; age when the ACE indicators and covariates were assessed; mean = 15.2, SD = 1.7). Notably, ethnicity superseded race such that those classified as White or Black are non-Hispanic, while Hispanic youth may be White or Black, in keeping with FDJJ protocols. The age at which the youth was first arrested was included, classifying youth as under 13, 13–14, 15, 16, or over 16 at age of first arrest (higher values indicated an older age at first arrest). Of note, 35% of the youth were first arrested under the age of 13.

### 2.3. Analytic Strategy

The current study employs secondary data analysis, utilizing a variety of multivariable methods as the study design. The analytic strategy undertaken included two steps to address our main research questions. First, a latent class analysis (LCA) [30,31] was used to identify the number of underlying typologies, or classes, present within the sample of youth. Specifically, LCA was used to distinguish between distinct subgroups of youth based on the categories of individuals who were victimized by the youth in the sample (e.g., strangers, family, authorities, etc.). The optimal number of classes was selected based on a variety of statistical fit indices including the Bayesian information criterion (BIC) [32], the sample-size adjusted BIC (ABIC) [33], the Lo–Mendell–Rubin adjusted likelihood ratio test (LRT) [34], the parametric bootstrapped likelihood ratio test (BLRT) [35], and entropy [36]. In these models, superior fit is indicated by lower values on the BIC and ABIC, as well as higher levels of entropy. Finally, model fit and the final class solution was selected based on substantive meaning and analytic utility (e.g., the relative size and distinctiveness of each group) [35]. 

Following the LCA, the estimated posterior probabilities were used to assign each youth to a class characterized by a specific combination of victim types. A series of multinomial logistic regression analyses were estimated using this classification, in which the latent classes became the outcomes modeled. The goal of this second portion of the analysis was to determine which youth characteristics and specific ACE exposures were associated with class membership. Finally, based on the importance of considering cumulative traumatic exposure, an additional multinomial logistic regression model was estimated to determine whether a count of traumatic exposure types (the ACE score) was significantly associated with class membership.

## 3. Results

Table 1 presents the percentage of the sample for each categorical measure, and the mean and standard deviations for all continuous measures, as well as the range (minimum and maximum) for all measures. As shown, the sample was, on average, 15 years of age when independent and control measures were established, 94.8% male, 48.5% Black, and 12.6% Hispanic. The average age of first arrest was approximately 15 years of age, while the average number of total victims for a sample youth was 1.44, with a range of having only 1 victim to a youth having 51 victims.

### 3.1. Identification of Latent Class Solution

LCA models were fit using MPlus Version 8 [37], with two to six latent classes specified. The fit statistics for each of the models are presented in Table 2. For each additional class between two and six, there were sizeable decreases in the BIC and ABIC. Additionally, the LMR, LRT, and BLRT suggested that the more complex models were a better fit to the data. Results of a seven-class solution suggested that adding an additional group led to a drop in classification specificity, identified by a drop in entropy as well as a small reduction in the BIC and a class consisting of very few youth (<2%). As such, the six-class solution was selected as the best fitting model to be assessed using multinomial logistic regression.

### 3.2. Victim Associations among Classes of Youth with Violent Sexual Offending

The item-response probabilities for all classes are shown in Table 3. The first class (labeled “Schoolmate victims”), constituting 18% of the sample, almost exclusively committed violent sexual offenses against classmates. The smallest class, Class two (6.4%; labeled “Stranger Victims”) was characterized by having committed offenses which predominately targeted strangers. The third class (labeled “Acquaintance Victims”), 31.3% of the sample, was the largest class and had committed violent sexual acts against acquaintances almost exclusively. The fourth class offended against siblings nearly exclusively, had the fewest average total victims (1.26), and composed 18.1% of the sample. The fifth class (12.9%, labeled “Diverse Victims”) was the only class with a mix of victim types with neighbors, intimate partners, and close friends being the predominate relationships, though having the highest number offending against parents/guardians and the youth’s own child of any class. Finally, the sixth class (13.2%; labeled “Other Relative Victims”) targeted other relatives (not parents/guardians, siblings, or the youth’s own child) and evidenced the highest average number of victims (1.59).

### 3.3. Covariates of Latent Class Membership

Upon identifying the six distinct latent classes of youth based on their relationship to their victims, the relationships between class membership and each of the individual-level covariates, including specific ACE exposures, were examined. Results presented in Table 4 were derived from a multinomial logistic regression model which assessed the association present between a given covariate and class membership. Estimates are shown in the form of relative risk ratios and reflect the odds of membership in the target class relative to the Acquaintance Victims class (Class 3) given a one-unit increase in a given covariate.

The results shown in Table 4 suggest that several measures have consistent relationships with class membership. Older youth were less likely to belong to the Schoolmate, Stranger, and Sibling classes (classes 1, 2, and 4) in comparison to the reference Acquaintance class. Males were more likely to target strangers and siblings than acquaintances, while Black youth were more likely to target classmates and strangers, but less likely to target siblings or have diverse victims, than to target acquaintances. Youth with child welfare history were more likely to target siblings, but less likely to target classmates or have diverse victims, than to target acquaintances. Exposure to community violence increases the odds of targeting strangers relative to acquaintances. With respect to individual ACE indicators, household substance abuse was the only ACE to increase the likelihood of having diverse victims (class 5), while physical abuse, sexual abuse, emotional neglect, and parental separation/divorce increased the likelihood of targeting siblings in comparison to acquaintances (while physical neglect decreased that likelihood).

Moving to including cumulative trauma (the ACE score) rather than individual ACE indicators, the results shown in Table 5 show the adjusted association between cumulative ACEs and latent class membership (including all control measures explored in Table 4). Youth with higher ACE exposures were less likely to target classmates (class 1) relative to acquaintances. Alternatively, higher ACE scores increased the likelihood of violent sexual offending against siblings and against other relatives (not parents/guardians, siblings, or the youth’s own child) relative to acquaintances.

## 4. Discussion

The current study findings further demonstrate the heterogeneity among juveniles who engage in sexual offending, in keeping with a growing body of prior work [3,4,5,6,7,8,9,10,11]. Importantly, this study advances from prior work in that we examine a large sample of over 5500 juveniles, all of which engaged in violent sexual felony offending. Further, the current study illustrates important distinctions between these youth based on the types of victims they target in their sexual offending. Additionally, the results suggest the extent of overall traumatic exposure, as well as different specific ACE exposures are associated with an increased likelihood of specific offender–victim relationships. Specifically, higher ACE exposure increases the likelihood a youth will violently victimize siblings and other relatives (not parent/guardian, siblings, or the youth’s own child), while decreasing the likelihood of targeting classmates in comparison to targeting acquaintances. Additionally, the current study shows specific ACE indicators additionally distinguish between the types of victims that youth may target, based on the relationship between those youth and their victims. Notably, among specific ACE indicators, only a history of household substance abuse increased the likelihood of having a more diverse groups of multiple types of victims. The strongest association between an individual ACE indicator and class membership was between sexual abuse history and targeting one’s own siblings. This is in keeping with a host of prior work indicating the importance of sexual abuse history in juvenile sexual offending [14], as well as recent work demonstrating the role of sexual abuse in distinguishing victims of nonsexual violent offending [21].

As discussed, recent work has examined the association between ACE exposure and victim types of nonsexual violent offending [21]. The current study advanced that approach to examining violent sexual offending. Both studies indicated that ACE exposures are relevant indicators in distinguishing youth who engage in violent offending with respect to the victims of that offending, based on the relationship between the offending youth and their victims. However, the association between cumulative trauma and specific ACEs with the types of victims varies across the two studies. For both nonsexual and sexual violent offending, cumulative ACE exposure increases the likelihood of victimizing family members. While household mental health problems and incarceration histories were highly relevant in distinguishing nonsexual violent offending victims [21], those exposures had little relevance for distinguishing sexual offending victims in the current study (with the exception of household incarceration decreasing likelihood of victimizing classmates). Alternatively, sexual abuse histories increased the likelihood of victimizing at least one type of family member in both studies. Future work should continue to examine the influence of cumulative trauma and specific exposures on the types of victims of violent offending, both sexual and nonsexual.

The results of the current study have prevention, intervention/treatment, and policy implications. Prevention of childhood maltreatment remains the primary defense against the deleterious effects of such exposure [38]. However, working with the study population of adolescents involved in the juvenile justice system requires intervention and treatment beyond prevention, due to the heightened traumatic exposure of justice-involved youth [24], and those with sexual offending specifically [15,28]. As such, universal screening for ACE exposure among all youth involved in the justice system and subsequent assessment of clinical symptomology of those with indicated ACEs becomes critical. Both specific ACE exposures and cumulative trauma matters with respect to likely future victims. Further, prior work indicates heightened ACE exposure increases recidivism [39]. While association among ACE and sexual reoffending is lacking, and rates of sexual recidivism among juveniles is low [1], the linkage of traumatic exposure to crime and delinquency is clear. Traumatic exposure is argued to have neurodevelopmental implications in negatively impacting essential self-regulatory behavioral and emotional responses [40,41], affecting regulation and social attachment [42], which increases the adoption of high-risk coping strategies and behaviors [43]. As such, treatment approaches should have both a trauma-specific component as well as potentially a multisystemic family-therapy component for those with ACEs indicated as increasing the likelihood of targeting family members. For those with exposures indicating the targeting of victims outside of the family, anger management and skill-building components, as well as violence prevention and dating violence-reduction interventions may be warranted. Of note, these potential recommendations should not replace traditional treatment related to sexual offending, but may be useful in reinforcing such treatment.

Certainly, the current study is not without limitation. Specifically, while exposure to each ACE indicator was known, data did not allow for uncovering the relationship between the abuser and the victimized youth (e.g., we know which youth had sexual abuse histories, but we do not know who sexually abused the youth). Presumably, the relationship of the abuser to the youth may have implications in the types of individuals that youth then violently abuse him/herself. Further, while those administering the assessment had access to official child welfare system records, the available data did not permit our examining of the proportion of abuse (physical or sexual) exposures that were reported to authorities, nor whether such abuse was substantiated, or whether the abuser was charged or convicted. Additionally, the common critique of the ACE score is it not being inclusive of exposures that occur outside of the home/family, such as community disadvantage, experiencing systemic racism, and exposure to community violence [44]. However, while not included as an ACE per se, the current study included both witnessing violence in the community as well as official child welfare system histories as independent measures. Finally, and critically important, is that the current study only included violent sexual offenses that became known to police and includes no information on those offenses which went unreported. Using the PACT full assessment oversamples higher risk youth, and includes approximately 32% of all youth referred to the FDJJ during the study timeframe [24,25], with included youth being older, higher proportions being male, greater proportions being Black, and less Hispanic. While the current study may not be as generalizable to all juveniles with sexually related offenses, it is generalizable to the most policy-relevant group, i.e., higher risk juveniles committing violent sexual offenses.

## 5. Conclusions

Juveniles who commit sexual offenses are a heterogeneous group. The current study examined typologies of juveniles who had been adjudicated for violent sexual offenses by age 18 with respect to the relationships between those youth and their victim(s). Importantly, the current study considered all known victims across all violent sexual offenses each youth committed. Our results suggest that specific traumatic exposures, as well as a measure of cumulative childhood trauma, have nuanced associations with violent sexual offending across different constellations of victim types. Heightened traumatic exposure increased the likelihood of victimizing siblings as did sexual abuse, physical abuse, history of child welfare placements, emotional neglect, and parental separation/divorce. Only household substance abuse increased the likelihood of violent sexual offending against diverse victim groups. Childhood adversity influences who a juvenile will violently sexually offend against.

## Figures and Tables

**Table 1:** Descriptive statistics for the analysis of the victims of juvenile sex offenders.

	Mean	SD	Min	Max
Victim Associations				
Stranger	0.097	0.447	0	9
Acquaintance	0.459	0.908	0	17
Schoolmate	0.264	0.715	0	11
Program Youth	0.014	0.195	0	10
Neighbor	0.087	0.456	0	10
Close Friend	0.044	0.735	0	51
Partner	0.052	0.223	0	1
Guardian	0.005	0.072	0	1
Sibling	0.181	0.385	0	1
Child	0.002	0.044	0	1
Other Relative	0.240	0.811	0	20
Total Victims	1.44	1.82	0	51
Youth Characteristics				
Age	15.23	1.67	9	20
Male	0.948	-	0	1
White	0.412	-	0	1
Black	0.485	-	0	1
Hispanic	0.126	-	0	1
Age at First Offense	2.08	1.1	1	5
History of Children Family Services	0.201	-	0	1
Mental Health Problems	0.206	-	0	1
Current Alcohol Use	0.104	0.373	0	2
Current Drug Use	0.251	0.583	0	2
Peer Associates	0.123	0.415	0	2
Temperament Index	0	0.681	−1	3
Exposure to Community Violence	0.420	-	0	1
Individual ACEs				
Emotional Abuse	0.206	-	0	1
Physical Abuse	0.211	-	0	1
Sexual Abuse	0.146	-	0	1
Emotional Neglect	0.197	-	0	1
Physical Neglect	0.113	-	0	1
Family Violence	0.385	-	0	1
Household Substance Abuse	0.131	-	0	1
Household Mental Health Problems	0.058	-	0	1
Divorce	0.851	-	0	1
Household Incarceration History	0.461	-	0	1
Cumulative ACE Score	2.76	1.94	0	10

Note: ACE = Adverse Childhood Experiences; SD = Standard Deviation.

**Table 2:** Latent class analysis model fit information.

Model	BIC	ABIC	Entropy	LMR LRT (*p*)	BLRT (*p)*
2-class	30,186.99	30,266.16	0.970	0.000	0.000
3-class	28,356.71	28,161.51	0.981	0.000	0.000
4-class	27,775.07	27,625.72	0.989	0.000	0.000
5-class	26,790.29	26,602.81	0.994	0.000	0.000
6-class	26,281.655	26,056.04	0.999	0.170	0.000

BIC = Bayesian information criterion; ABIC = Sample size adjusted BIC; LMR LRT = Lo–Mendell–Rubin adjusted likelihood ratio test; BLRT= Parametric bootstrapped likelihood ratio test.

**Table 3:** Description of victim associations among youth.

	Class 1: Schoolmate Victims *n* = 999	Class 2: Stranger Victims *n* = 355	Class 3: Acquaintance Victims *n* = 1735	Class 4: Sibling Victims *n* = 1004	Class 5: Diverse Victims *n* = 715	Class 6: Other Relative Victims *n* = 731
Total Victims	1.496	1.459	1.495	1.262	1.361	1.590
Stranger	0.000	1.383	0.020	0.011	0.000	0.000
Acquaintance	0.053	0.000	1.384	0.090	0.000	0.000
Schoolmate	1.419	0.034	0.000	0.019	0.000	0.018
Program Youth	0.003	0.003	0.001	0.004	0.098	0.001
Neighbor	0.010	0.008	0.022	0.023	0.557	0.012
Close Friend	0.003	0.011	0.006	0.004	0.306	0.001
Partner	0.005	0.000	0.010	0.007	0.358	0.005
Guardian	0.003	0.000	0.000	0.004	0.029	0.001
Sibling	0.000	0.000	0.000	1.000	0.000	0.000
Child	0.000	0.000	0.000	0.002	0.013	0.000
Other Relative	0.000	0.020	0.052	0.099	0.000	1.550

**Table 4:** Multinomial logistic regression predicting class membership.

	Class 1: Schoolmate Victims *n* = 999		Class 2: Stranger Victims *n* = 355		Class 4: Sibling Victims *n* = 1004		Class 5: Diverse Victims *n* = 715		Class 6: Other Relative Victims *n* = 731	
	RRR/95% CI		RRR/95% CI		RRR/95% CI		RRR/95% CI		RRR/95% CI	
Age	0.919 **	0.869–0.971	0.837 ***	0.768–0.913	0.835 ***	0.788–0.884	1.047	0.982–1.118	0.962	0.904–1.023
Male	0.927	0.650–1.321	2.926 **	1.336–6.411	2.019 ***	1.369–2.979	0.994	0.673–1.469	0.973	0.672–1.409
Black	2.213 ***	1.832–2.673	1.832 ***	1.390–2.415	0.537 ***	0.447–0.646	0.660 ***	0.540–0.807	1.122	0.921–1.367
Hispanic	1.832 ***	1.394–2.408	1.796 **	1.225–2.634	0.888	0.679–1.159	1.130	0.859–1.487	1.520 **	1.148–2.012
Age at First Offense	0.882 **	0.807–0.964	1.288 ***	1.137–1.459	1.100 *	1.005–1.203	1.097 *	1.003–1.200	1.011	0.921–1.110
History of Children Family Services	0.735 *	0.572–0.944	1.003	0.711–1.415	1.469 ***	1.184–1.824	0.748 *	0.570–0.980	1.096	0.854–1.406
Mental Health Problems	1.001	0.799–1.252	0.706 *	0.502–0.994	0.997	0.809–1.229	1.031	0.814–1.306	0.975	0.767–1.240
Current Alcohol Use	1.085	0.853–1.381	1.231	0.908–1.668	0.877	0.666–1.154	0.879	0.680–1.136	0.937	0.693–1.266
Current Drug Use	0.999	0.861–1.160	1.111	0.905–1.363	0.660 ***	0.548–0.795	0.926	0.784–1.094	0.697 ***	0.573–0.848
Peer Associations	0.938	0.778–1.130	0.923	0.713–1.197	0.711 **	0.558–0.906	1.017	0.828–1.249	0.642 **	0.489–0.843
Temperament Index	0.966	0.830–1.123	1.177	0.950–1.458	0.781 **	0.671–0.910	0.957	0.809–1.131	0.738 ***	0.622–0.876
Exposure to Community Violence	0.978	0.828–1.156	1.350 *	1.062–1.716	0.867	0.730–1.030	1.167	0.970–1.403	0.936	0.778–1.126
Emotional Abuse	1.389 *	1.009–1.911	0.908	0.579–1.425	1.012	0.771–1.328	1.102	0.802–1.515	1.170	0.853–1.606
Physical Abuse	1.099	0.822–1.468	0.975	0.645–1.473	1.482 **	1.144–1.920	1.297	0.967–1.740	1.057	0.784–1.425
Sexual Abuse	0.614 **	0.444–0.850	0.871	0.562–1.348	2.022 ***	1.598–2.558	0.831	0.611–1.130	1.205	0.906–1.604
Emotional Neglect	1.076	0.864–1.341	1.363 *	1.008–1.844	1.258 *	1.015–1.560	1.201	0.947–1.523	1.176	0.926–1.494
Physical Neglect	0.840	0.609–1.160	0.905	0.579–1.414	0.677 **	0.515–0.889	0.879	0.634–1.218	0.954	0.701–1.300
Family Violence	0.763	0.562–1.035	0.905	0.592–1.385	1.287	0.989–1.676	0.964	0.711–1.308	1.081	0.802–1.459
Household Substance Abuse	1.036	0.794–1.350	0.762	0.505–1.150	0.986	0.765–1.271	1.428 **	1.096–1.862	1.176	0.895–1.547
Household Mental Health Problems	1.019	0.708–1.468	1.031	0.604–1.762	0.866	0.612–1.226	0.704	0.465–1.068	1.301	0.910–1.860
Divorce	0.766 *	0.616–0.952	1.015	0.726–1.419	2.104 ***	1.596–2.773	0.920	0.723–1.171	1.078	0.837–1.389
Household Incarceration History	0.826 *	0.696–0.981	0.898	0.701–1.150	0.886	0.744–1.055	0.913	0.753–1.105	1.035	0.857–1.250
Constant	2.641 *	1.132–6.159	0.407	0.099–1.682	2.071	0.851–5.044	0.199 **	0.073–0.542	0.618	0.241–1.584

Note: Group 3 (Acquaintance victims) represents the base outcome; * *p* < 0.05, ** *p* < 0.01, *** *p* < 0.001; RRR = Relative Risk Ratio; CI = Confidence Interval.

**Table 5:** Association between ACE Score & Victim Class Membership.

	Association between Cumulative ACE Score and Latent Class Membership	
	RRR/95% CI	
Class 1: Schoolmate Victims	0.917 *	0.869–0.968
Class 2: Stranger Victims	0.943	0.872–1.019
Class 3: Acquaintance Victims	Reference Category	
Class 4: Sibling Victims	1.206 **	1.147–1.268
Class 5: Diverse Victims	1.027	0.970–1.087
Class 5: Other Relative Victims	1.106 **	1.045–1.170

Note: Results drawn from a multinomial logistic regression analysis. Model includes all previously mentioned control variables. Group 3 (Acquaintance victims) represents the base outcome; ACE = Adverse Childhood Experience; RRR = Relative Risk Ratio; CI = Confidence Interval; * *p* < 0.01, ** *p* < 0.001; *n* = 5538.

## Data Availability

The data that support the findings of this study are available from the Florida Department of Juvenile Justice (data source), but restrictions apply to the availability of these data, which were used under license for the current study, and thus are not publicly available.
